# Supplementary material for: The geographical maldistribution of obstetricians and gynecologists in Japan
Source: PLoS One. 2021 Jan 12;16(1):e0245385. doi: 10.1371/journal.pone.0245385 (PMC7802964; doi:10.1371/journal.pone.0245385)
Supplement: Attachment — Submitted filename: Response to reviewers.docx [file pone.0245385.s001.docx]

Response to reviewer

We wish to express our appreciation to the Reviewer for his or her insightful comments, which have helped us significantly to the paper.

Comment:

L101 Do you mean you were permitted by the Ministry of Health to use the individualized data for the research purpose? If so, you need to clearly state so and add the permission number issued by the Ministry.

Response:

Yes. We added the following sentence.

L102

Under the permission of the Ministry of Health, Labour and Welfare (Seito-0408-1), we obtained anonymized individual data from this census.

Comment:

L171 The word “significant” should be avoided because statistical test for the difference is not conducted (if you done, the results would've been much more persuasive..).

Response:

We deleted the word “significant”.

L173

The Gini coefficient for obstetricians/gynecologists was stable for the period between 2002 to 2006, began to rise in 2008, and continued to rise until 2018.

Comment:

L208-215 It is utterly unscientific that you don’t mention the huge gap of Gini coefficient between male >40 and female >40 yo. It looks like “the elephant in the room”, which is far more important than the trend of the coefficient itself.

Response:

We added sentences in Result section and Discussion section.

L214

The Gini coefficients for males and females aged 40 years and above were not considerably different, however, the Gini coefficient for females under 40 years was considerably higher than that of males under 40 years. In addition,

L230

Therefore, the Gini coefficient of females aged 40 years and above was considerably higher than that of males aged 40 years and above.

Comment:

L220 The absolute value of coefficient itself (0.400) is not so meaningful because it changes substantially depending on the variables used and geographic unit of analysis.

Response:

We also know the magnitude of the Gini coefficient itself is not so meaningful. So, we deleted the absolute value.

L225

The results of this study showed that the maldistribution of obstetricians/gynecologists was worse compared to that of other physicians or pediatricians.

Comment:

L220 “might” should be “potentially”

Response:

Thank you. We changed the sentence.

L226

An increase in the number of female obstetricians/gynecologists have potentially contributed to this since female physicians tended to be more unevenly distributed compared to their male counterparts.

Comment:

L260- You might consider the effect of the nature of OBGY practice on geographic distribution. (Tohoku J Exp Med 2020 May;251(1):1-8. doi: 10.1620/tjem.251.1.) Also please pay attention to the “selection and concentration” of obstetricians into urban high-volume hospitals, which is proposed and facilitated forcefully both by the government and the Japan OBGY Association. (Int J Health Geogr. 2016 Jan 22;15:4. doi: 10.1186/s12942-016-0035-y; J Obstet Gynaecol Res. 2015 Jun;41(6):919-25. doi: 10.1111/jog.12663. )

Response:

We added the following sentences and 2 more references.

L269

In addition, the worsening uneven distribution of obstetricians/gynecologists is considered to be largely influenced by the government and Academic Societies that have promoted the selection and concentration of obstetric hospitals [21, 22]. If the obstetrics/gynecology workforce continues to decline, it will force individual doctors in the facilities that handle delivery to overwork, and concurrently lead to a decline in the quality of obstetric care. Therefore, the government and the Japan Society of Obstetrics and Gynecology have promoted the selection and concentration of obstetric hospitals. As a result, the uneven distribution of regions in each municipality may have increased. Furthermore, it is considered that the uneven distribution of obstetricians and obstetricians and gynecologists has further expanded due to the increase in the proportion of female doctors in parallel with this movement. The concentration is aimed at improving the working environment and ensuring a sustainable perinatal medical system, but it may also worsen access to delivery facilities in rural areas. It will become even more important to develop the working environment to enable the complete utilization of the skills of increasing number of female physicians.
